# Orphan nuclear receptor NR4A2 induces transcription of the immunomodulatory peptide hormone prolactin

**DOI:** 10.1186/s12950-015-0059-2

**Published:** 2015-02-18

**Authors:** Joseph M McCoy, Dana E Walkenhorst, Keegan S McCauley, Hiba Elaasar, Jordan R Everett, Kimberlee S Mix

**Affiliations:** Department of Biological Sciences, Loyola University New Orleans, New Orleans, Louisiana 70118 USA

**Keywords:** Orphan nuclear receptor, Transcription, Prolactin, Arthritis, Synoviocytes, Promoter

## Abstract

**Background:**

Nuclear receptor 4A2 (NR4A2) is an orphan nuclear receptor and constitutively active transcription factor expressed at elevated levels in inflamed joint tissues from patients with arthritis. Inflammatory mediators rapidly and potently induce NR4A2 expression in resident joint cells and infiltrating immune cells. This receptor promotes synovial hyperplasia by increasing proliferation of synoviocytes and inducing transcription of matrix degrading enzymes and pro-inflammatory mediators. In order to further elucidate the molecular mechanisms of NR4A2, we conducted a gene expression screen to identify novel transcriptional targets of NR4A2 that may contribute to arthritis progression.

**Methods:**

NR4A2 was over-expressed in human synoviocytes by lentiviral transduction and gene expression changes were measured using qPCR arrays specific for inflammation, proliferation, adhesion, and migration pathways. Subsequent analysis focused on the most potently induced gene prolactin (PRL). Messenger RNA levels of PRL and PRL receptor (PRL-R) were measured by RT-qPCR and protein levels were measured by ELISA. PRL promoter studies were conducted in synoviocytes transiently transfected with NR4A2 and PRL reporter constructs. Molecular responses to PRL in synoviocytes were addressed using qPCR arrays specific for JAK/STAT signaling pathways.

**Results:**

PRL was the most potently induced gene on the qPCR arrays, exhibiting a 68-fold increase in response to ectopic NR4A2. This gene encodes an immunomodulatory peptide hormone with roles in autoimmune diseases and inflammation. Induction of PRL mRNA and secreted protein by NR4A2 was confirmed in subsequent experiments, with increases of 300-fold and 18-fold respectively. Depletion of endogenous NR4A receptors with shRNA reduced basal and PGE_2_-induced PRL levels by 95%. At the transcriptional level, NR4A2 requires a functional DNA binding domain to transactivate the distal PRL promoter. Deletional analysis indicates that NR4A2 targets a region of the distal PRL promoter spanning −270 to -32 bp. In synoviocytes, recombinant PRL regulates several genes involved in inflammation, proliferation, and cell survival, suggesting that NR4A2 induced PRL may also impact these pathways and contribute to arthritis progression.

**Conclusions:**

These results provide the first evidence for transcriptional regulation of the immunomodulatory peptide hormone PRL by NR4A2 in synoviocytes, and highlight a novel molecular pathway in inflammatory arthritis.

**Electronic supplementary material:**

The online version of this article (doi:10.1186/s12950-015-0059-2) contains supplementary material, which is available to authorized users.

## Background

Inflammatory arthritis is driven by pro-inflammatory mediators such as tumor necrosis factor-α (TNF-α) and prostaglandin E_2_ (PGE_2_) that induce synovial hyperplasia and trigger the erosion of cartilage and bone. During the progression of arthritis, fibroblast-like synoviocytes transform into aggressive cells that proliferate rapidly, grow in an anchorage-independent manner, and invade adjacent cartilage and bone [1]. At the molecular level, transformed synoviocytes exhibit altered gene expression profiles that reflect an increased capacity to degrade extracellular matrix, promote inflammation, and stimulate angiogenesis [2]. These processes are tightly controlled by transcription factors such as nuclear factor kappa B (NFκB), cAMP response element binding (CREB), c-Myc, and p53 [3-6]. In order to develop innovative strategies to reduce inflammation and preserve joint integrity in patients with arthritis, it is important to further elucidate the transcriptional networks and signaling pathways active in arthritis.

The orphan nuclear receptor 4A2 (NR4A2/NURR1, nuclear receptor related-1) has recently emerged as a novel transcription factor with regulatory roles in inflammatory arthritis [7-13]. In contrast to most members of the nuclear receptor super-family, NR4A2 is a ligand-independent transcription factor whose activity is tightly controlled at the level of expression and post-translational modification [14]. NR4A2 is over-expressed in inflamed synovial tissues and cartilage from patients with rheumatoid arthritis (RA), osteoarthritis (OA), and psoriatic arthritis (PsA), and NR4A2 is the most highly expressed member of the NR4A receptor family in these tissues [7-12]. Inflammatory cytokines and prostaglandins rapidly and potently induce the nuclear expression of NR4A2 in synoviocytes, chondrocytes, endothelial cells, and immune cells [7,9,11,15]. While the molecular functions of NR4A2 are not yet fully understood, NR4A2 may serve as a viable drug target to regulate inflammatory pathways. In fact, conventional therapies such as methotrexate, dexamethasone, and 6-mercaptopurine can modulate NR4A2 expression and activity [8,11,16], suggesting that some of the clinical benefits of these agents may be mediated through effects on NR4A2. Furthermore, novel small molecules that specifically target NR4A2 may provide new strategies to treat inflammatory arthritis [17].

We have recently demonstrated that NR4A2 promotes synovial hyperplasia by increasing synoviocyte proliferation, anchorage-independent growth, and invasion through type II collagen [10]. In line with these cellular changes, NR4A2 induces transcription of the cartilage degrading enzyme, MMP-13, and decreases expression of the tissue inhibitor of MMPs, TIMP-2 [10]. Additional transcriptional targets of NR4A2 in synoviocytes include IL-8, amphiregulin, and Kit ligand [13,18], indicating that NR4A2 converges on transcriptional pathways that control cell recruitment, proliferation, and tissue degradation. However, the transcriptional mechanisms of NR4A2 have not been fully elucidated, and this receptor likely regulates several genes that contribute to inflammation and joint degradation in arthritis.

In this study, we conducted a gene expression screen to identify novel transcriptional targets of NR4A2 that may contribute to disease progression. Interestingly, the immunomodulatory peptide hormone prolactin (PRL) emerged as the most potently induced gene among a panel of genes associated with inflammation, proliferation, adhesion, and migration. While PRL has fundamental roles in reproduction and development, this pleiotropic hormone also contributes to autoimmune diseases and inflammation [19]. In patients with RA, elevated levels of PRL correlate with disease activity and structural damage [19-21]. Furthermore, reducing systemic PRL levels with the dopamine agonist bromocriptine has beneficial effects in patients with RA and in animal models of disease [22]. Given the clinical links between PRL levels and RA, we were intrigued by the possibility that NR4A2 could be a potent regulator of this immunomodulatory hormone.

Circulating PRL is largely derived from the anterior pituitary gland, however it is also produced locally in inflamed joints. Synoviocytes, chondrocytes, and infiltrating immune cells synthesize PRL and these cells may contribute to elevated levels of PRL in synovial fluid from patients with RA [20,23-26]. PRL regulates synoviocyte proliferation, chondrocyte survival, and the expression of MMPs and inflammatory cytokines by engaging cell surface PRL receptors and activating intracellular JAK/STAT signaling pathways [23,25,27]. The transcriptional mechanisms supporting PRL synthesis within inflamed joints are largely unexplored and we hypothesized that NR4A2 may serve as a critical transcription factor involved in this process.

After identifying PRL as a putative NR4A2 target gene, we confirmed that NR4A2 potently induces PRL mRNA and protein expression in human synoviocytes. Depletion of the NR4A receptors by shRNA abrogates PGE_2_-induced PRL expression, suggesting that these receptors are critical for PRL synthesis. At the transcriptional level, NR4A2 targets the proximal region of the distal PRL promoter through a mechanism that requires DNA binding. Recombinant PRL regulates gene expression pathways that control inflammation, proliferation, and cell survival in synoviocytes, suggesting that NR4A2-induced PRL may elicit similar autocrine responses that may subsequently impact arthritis progression. Our results provide the first evidence for transcriptional regulation of PRL by the orphan receptor NR4A2 in synoviocytes, and highlight a novel molecular pathway in inflammatory arthritis.

## Methods

### Cell culture

K4IM normal human synoviocytes were cultured in RPMI 1640 medium with 10% FBS as described previously [7,28]. Stable NR4A-depleted K4IM cells, kindly provided by Dr. Evelyn Murphy (University College Dublin), were generated by lentiviral transduction of control scrambled short hairpin RNA (shRNA) and shRNA specific for NR4A1, NR4A2, and NR4A3 receptors [10]. Treatments were conducted in serum-free RPMI medium. Human fibroblast-like synoviocytes (HFLS) derived from normal and RA synovial tissues were obtained from Cell Applications, Inc. HFLS were cultured in synoviocyte growth medium and transferred to basal medium for treatments (Cell Applications, Inc.). PGE_2_ (Sigma-Aldrich) was used at a final concentration of 1 μM in all experiments. Recombinant human TNF-α (rhTNF-α, Sigma-Aldrich) was used at a final concentration of 10 ng/mL and rhPRL (R & D Systems) was used at concentrations ranging from 10 ng/mL to 1 μg/mL. Cell proliferation and viability was measured using an MTT based non-radioactive assay (Promega).

### Lentiviral transductions

Lentiviral particles containing empty vector control or CMV-NR4A2 cDNA were produced in 293FT cells using the ViraPower Lentiviral Expression System (Invitrogen). Plasmids (pLenti-CMV, pLenti-CMV-hNR4A2) were kindly provided by Dr. C.J.M de Vries (University of Amsterdam). HFLS and K4IM cells were cultured in growth media as described above to 50% confluence in 6 well plates. Cells were transduced in a total volume of 1 mL consisting of a 50:50 mix of growth media and filtered lentiviral particles. Following a 12-hour incubation with lentiviral particles, cells were washed and transferred to serum free media for 48 hours.

### RNA extraction, reverse transcription, and PCR

Total RNA from synoviocytes was isolated at time-points indicated in figure legends using TRIzol reagent (Sigma-Aldrich). RNA was reverse-transcribed into cDNA using iScript Select cDNA synthesis kit with an oligo-dT primer (Bio-Rad). Standard qPCR was conducted using iQ Super Mix (Bio-Rad), TaqMan primers and probes (Life Technologies), and a Bio-Rad CFX96 qPCR machine. Relative expression levels were calculated using the 2^-ddCt^ method, normalizing to GAPDH. TaqMan Human Angiogenesis Fast Arrays were conducted in a 96-well format using TaqMan Universal Master Mix II (Life Technologies) and plates were run in an Applied Biosystems StepOnePlus 7500 Fast machine. Gene expression data was normalized to four housekeeping genes (18S, GAPDH, HPRT1, GUSB) and relative quantification analysis was conducted using DataAssist software (Applied Biosystems). Human JAK/STAT Signaling Pathway RT^2^ Arrays were conducted in a 96-well format using SYBR Green reagents (Qiagen). Gene expression data was normalized to three housekeeping genes (B2M, GAPDH, RPLP0) and relative quantification analysis was conducted using web-based PCR Array Data Analysis software (SABiosciences). Standard RT-PCR was used to detect the presence of exon 1A in PRL mRNA using primers in exon 1A and exon 2 described previously [29]. GAPDH was used as a housekeeping control. PCR products were separated on a 2% agarose 1xTAE gel, stained with ethidium bromide, and images were collected with a Gel Doc system (BioRad).

### ELISA

Conditioned media from K4IM transduction experiments were collected and stored at −80°C. Secreted PRL in 100 μl of each sample was quantified in triplicate using the DuoSet human PRL ELISA kit (R&D Systems). A standard curve was generated using rhPRL.

### Transfections

K4IM cells were transiently transfected in triplicate in 24 well plates using Geneporter 3000 reagents in 10% FBS RPMI 1640 media (DNA Therapy Systems). Human distal PRL promoter sequences (−3000, −270, and -32 bp) were cloned into pGL3-Basic and kindly provided by Dr. Birgit Gellersen (University of Hamburg) [30,31]. Cells were co-transfected with control CMV-β-galactosidase, CMX-NR4A2 (gift from Dr. Thomas Perlmann, Karolinska Institute), or CMX-NR4A2 C283G DNA binding domain mutant [9]. Cells were transferred to serum-free media 12 hours post-transfection and whole cell extracts were collected 24 hours later in reporter lysis buffer (Promega). Luciferase assay reagent (Promega) was combined with a 20 μl sample of each extract and luminescence was measured on a Promega GloMax 20/20 luminometer. Transfection experiments were repeated at least three times in triplicate and consistent results were obtained.

### Statistical analysis

Prism software was used to compute the Student’s t-test for all gene expression and transfection data. Results are presented as the mean +/− standard deviation values.

## Results

**Figure 1:**
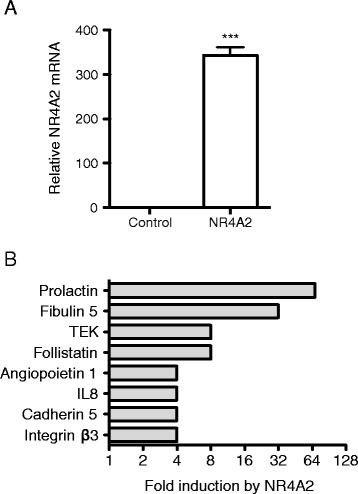
**NR4A2 induces expression of genes involved in synovial hyperplasia and inflammation. A**. K4IM human synoviocytes were transduced in triplicate with lentiviral particles containing control empty or CMV-NR4A2 expression vectors. RNA was harvested after 48 hours and NR4A2 expression was measured by RT-qPCR using GAPDH as a control. ***p < 0.001. **B**. Pooled triplicate cDNA from the same experiment was analyzed on TaqMan Human Angiogenesis qPCR arrays. Gene expression data was normalized to 4 housekeeping genes and the genes most highly induced by NR4A2 are displayed.

Previous studies have demonstrated that NR4A2 is over-expressed in inflamed synovial tissues from patients with RA and PsA, and inflammatory mediators potently induce NR4A2 expression in primary synoviocytes [8,10-13]. To address the impact of elevated levels of NR4A2 in synoviocytes, NR4A2 was ectopically expressed in the normal human synoviocyte cell line, K4IM. This cell line has been used to study synoviocyte responses to inflammation and NR4A2 expression patterns are consistent with those observed in primary human synoviocytes [10,13,18,28]. The related receptors NR4A1 and NR4A3 are expressed at lower levels in this cell line [10]. K4IM cells were transduced with an NR4A2 expression vector, and NR4A2 mRNA levels increased 350-fold after 48 hours (Figure 1A, p < 0.001). These levels are comparable to endogenous levels of NR4A2 induced by inflammatory cytokines [10,13]. We have previously demonstrated that ectopically expressed NR4A2 protein localizes to the nucleus where it functions as a constitutively active transcription factor [9,13]. To identify potential transcriptional targets of NR4A2, cDNA from control and NR4A2 transduced cells was compared on qPCR arrays. Expression levels of 92 genes involved in inflammation, cell proliferation, adhesion, and migration were surveyed (Additional file 1: Table S1). NR4A2 over-expression did not alter the expression of most genes detected on the arrays, indicating that this receptor does not have a global transcriptional effect. However, NR4A2 induced a subset of eight genes by 4-fold or more (Figure 1B). PRL was the most potently induced gene, with NR4A2 upregulating its expression by 68-fold. IL-8 was induced 4-fold, consistent with reports indicating that this chemokine is transcriptionally regulated by NR4A2 [13,18]. Additional genes induced by NR4A2 include fibulin 5, TEK, follistatin, angiopoietin-1, cadherin 5, and integrin β3. Interestingly, some of these genes have also been detected in inflamed joints and they modulate synoviocyte function, angiogenesis, and inflammation [32,33].

**Figure 2:**
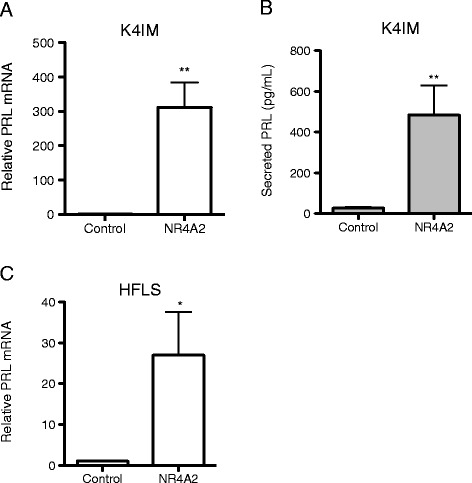
**NR4A2 induces PRL mRNA and protein secretion. A**. K4IM human synoviocytes were transduced in triplicate with lentiviral particles containing control empty or CMV-NR4A2 expression vectors. RNA was harvested after 48 hours and PRL expression was measured by RT-qPCR using GAPDH as a control. **p < 0.005. **B**. Conditioned media from transduced K4IM cells was collected after 48 hours and secreted PRL was measured by ELISA. **p < 0.005. **C**. HFLS derived from normal synovial tissue were transduced in triplicate with lentiviral particles containing control empty or CMV-NR4A2 expression vectors. RNA was harvested after 48 hours and PRL expression was measured by RT-qPCR using GAPDH as a control. *p < 0.01.

Since PRL was identified as the most highly induced gene on the qPCR arrays, and this peptide hormone has immunomodulatory roles in inflammatory joint diseases [19], we focused on PRL as a potential target gene of NR4A2. Induction of PRL mRNA was confirmed in subsequent experiments, with a potent 300-fold increase in response to ectopic NR4A2 in K4IM cells (Figure 2A, p < 0.005). Furthermore, levels of secreted PRL protein in conditioned media from these cells increased 18-fold, from 27 to 484 pg/mL (Figure 2B, p < 0.005). Regulation of PRL was also confirmed in primary human fibroblast-like synoviocytes (HFLS) derived from normal synovial tissue, where mRNA levels were induced 27-fold by ectopic NR4A2 (Figure 2C, p < 0.01).

**Figure 3:**
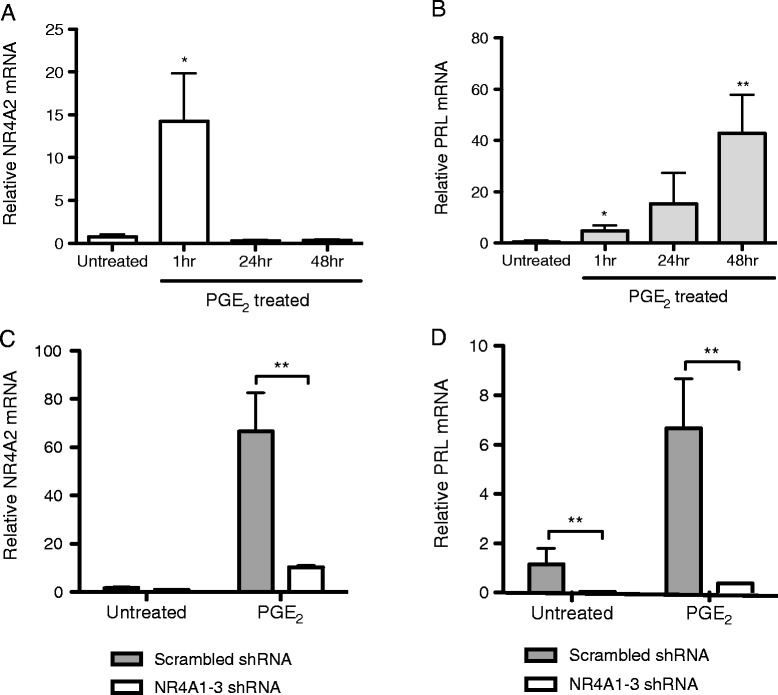
**NR4A receptors are required for PGE**_**2**_ **induction of PRL. A**. Triplicate wells of K4IM synoviocytes were left untreated or stimulated with PGE_2_ (1 μM) in serum-free media for the times indicated. NR4A2 expression levels were measured by RT-qPCR. *p < 0.05. **B**. PRL expression levels were measured by RT-qPCR in the same experiment. *p < 0.05, **p < 0.005. **C**. Scrambled shRNA or NR4A1-3 shRNA transduced K4IM synoviocytes were left untreated or treated with PGE_2_ for one hour. NR4A2 expression levels were measured by RT-qPCR. **p < 0.005. **D**. Scrambled shRNA or NR4A1-3 shRNA transduced K4IM synoviocytes were left untreated or treated with PGE_2_ for 24 hours. PRL expression levels were measured by RT-qPCR. **p < 0.005.

To address the impact of endogenous NR4A2 on PRL expression, K4IM cells were treated with a potent inducer of NR4A2 expression, PGE_2_ [7]. NR4A2 mRNA was induced 14-fold after 1 hour of stimulation, and levels returned to baseline by 24 hours (Figure 3A, p < 0.05). In contrast, PRL exhibited a delayed expression pattern in response to PGE_2_, with mRNA levels increasing from 4-fold to 43-fold in 48 hours (Figure 3B, p < 0.005). These kinetics suggest that the early induction of NR4A2 may subsequently contribute to PRL induction. To test this, K4IM cells were stably transfected with shRNA specific for NR4A1-3 or a scrambled control. NR4A2 levels were reduced by 85% in response to NR4A1-3 shRNA (Figure 3C, p < 0.005). In turn, basal and PGE_2_-induced PRL levels were reduced by 95% (Figure 3D, p < 0.005), indicating that the NR4A receptors are required for PRL expression.

**Figure 4:**
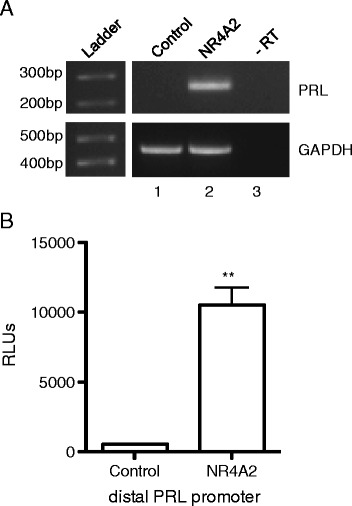
**NR4A2 transactivates the distal PRL promoter in synoviocytes. A**. RT-PCR was conducted with primers specific for exon 1A and exon 2 of PRL or GAPDH and products were resolved on an agarose gel. PRL primers yielded the expected product size of 275 bp, indicating the presence of exon 1A in PRL transcripts. GAPDH specific primers produced the expected product size of 451 bp. Lanes 1–3 contained the following PCR templates: cDNA from control transduced K4IM cells, cDNA from NR4A2 transduced K4IM cells, and negative control. **B**. K4IM synoviocytes were transiently transfected in triplicate with a distal PRL promoter (−3000 bp) luciferase reporter and control or NR4A2 expression vectors as indicated. Relative luciferase units (RLUs) were measured in cell lysates 48 hours post-transfection. **p < 0.005.

Since NR4A2 is a constitutively active transcription factor, we hypothesized that NR4A2 induces PRL expression at the level of transcription. PRL is regulated by two distinct promoters located on human chromosome 6. The proximal promoter confers expression in the pituitary, while the distal promoter, located approximately 5 kb upstream of the start-site of transcription, typically controls expression in extra-pituitary sites [34]. Transactivation of the distal promoter results in the incorporation of an additional noncoding exon 1A in the PRL transcript, while transcripts derived from the proximal promoter lack this sequence [35]. To determine which promoter is activated by NR4A2 in synoviocytes, we conducted RT-PCR with primers specific for exon 1A and exon 2 of PRL [29]. A product with the expected size of 275 bp was detected in cDNA from K4IM cells transduced with NR4A2, confirming the presence of exon 1A in PRL transcripts (Figure 4A). This finding indicates that NR4A2-induced PRL is in fact derived from the distal promoter. To extend this observation, transient transfections were conducted with a distal PRL promoter luciferase reporter (−3000 bp). Co-transfection of an NR4A2 expression vector potently induced transactivation of this promoter (Figure 4B, p < 0.005), confirming that NR4A2 targets the distal promoter to increase PRL transcription.

**Figure 5:**
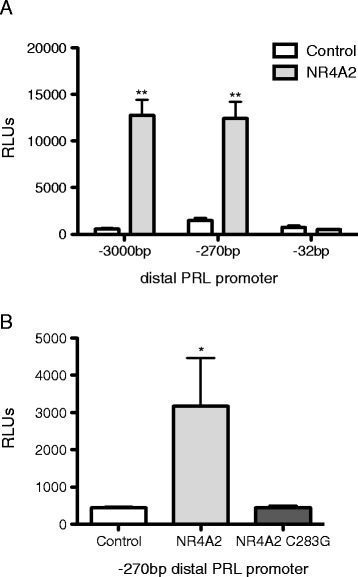
**DNA**-**binding domain of NR4A2 is required for transactivation of PRL promoter. A**. K4IM synoviocytes were transiently transfected in triplicate with distal PRL promoter luciferase reporters (−3000 bp, −270 bp, −32 bp) and control or NR4A2 expression vectors as indicated. Relative luciferase units (RLUs) were measured in cell lysates 48 hours post-transfection. **p < 0.005. **B**. K4IM synoviocytes were transiently transfected in triplicate with a distal PRL promoter (−270 bp) luciferase reporter and control, NR4A2, or NR4A2 C283G (DNA binding domain mutant) expression vectors as indicated. Relative luciferase units (RLUs) were measured in cell lysates 48 hours post-transfection. *p < 0.05.

NR4A2 can activate transcription through direct binding to NR4A-binding response elements (NBRE: AAAGGTCA) in the promoters of target genes [14]. However, analysis of the PRL promoter sequence did not identify any putative NBRE sites, suggesting that NR4A2 may interact with novel *cis*-elements to induce PRL expression. Deletional analysis of the distal PRL promoter was conducted to identify the site (s) targeted by NR4A2. The full-length -3000 bp and truncated -270 bp promoters are induced by NR4A2 to a similar extent (Figure 5A, p < 0.005), suggesting that this intervening sequence is not required for NR4A2 activity. In contrast, NR4A2 failed to transactivate the -32 bp promoter, indicating that NR4A2 *cis*-elements are absent in this truncated promoter. These results suggest that NR4A2 transactivates the PRL promoter by targeting sequences in the region spanning −32 to -270 bp. Furthermore, a well-characterized point mutation in the DNA binding domain of NR4A2, C283G [9], abrogated transactivation of the promoter, suggesting that direct DNA binding is required for PRL transcription (Figure 5B). Taken together, we have identified PRL as a novel transcriptional target of NR4A2 in synoviocytes and have determined that NR4A2 targets the distal promoter region spanning −32 to 270 bp through a mechanism that requires DNA binding.

**Figure 6:**
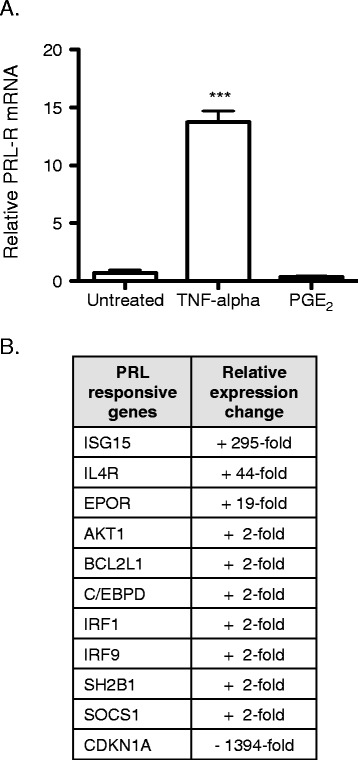
**PRL regulates expression of genes involved in inflammation**, **proliferation**, **and cell survival. A**. K4IM synoviocytes were treated in triplicate with TNF-α (10 ng/mL) or PGE_2_ (1 μM) for 24 hours. PRL-R was measured by RT-qPCR. ***p < 0.001. **B**. cDNA from K4IM synoviocytes treated with rhPRL (1 μg/mL, 24 hours) was analyzed on JAK/STAT signaling pathway qPCR arrays. Gene expression data was normalized to 3 housekeeping genes and the genes most highly regulated by rhPRL are displayed.

Next, we explored the functional impact of PRL expression in synoviocytes. Extracellular PRL binds to cell surface PRL receptors (PRL-R) and activates JAK/STAT signaling pathways. To address potential autocrine effects of PRL on synoviocytes, we first documented the expression levels of PRL-R. K4IM synoviocytes express detectable levels of PRL-R by RT-qPCR, suggesting that this receptor system is intact. Furthermore, TNF-α potently induces PRL-R expression by 15-fold, while PGE_2_ has no effect (Figure 6A), suggesting that inflammatory mediators may differentially modulate PRL signaling. Previous studies suggest that PRL can regulate proliferation and survival of RA synovial cells and chondrocytes [23,25,27], thus we investigated the impact of PRL on these processes. However, we did not observe any effects on the proliferation or viability of K4IM synoviocytes or primary HFLS derived from RA synovial tissue in response to recombinant human PRL (Additional file 2: Figure S1).

To further address the impact of PRL on synoviocytes, we surveyed global gene expression changes using qPCR arrays specific for the JAK/STAT pathways (Additional file 3: Table S2). Importantly, JAK/STAT signaling cascades are activated by PRL, and the selected qPCR arrays contain genes that are known targets of PRL. A group of 11 genes were significantly altered by rhPRL treatment (Figure 6B). Importantly, Akt1, IRF1, and SOCS-1 are transcriptional targets of PRL [36-38], and we confirmed a 2-fold increase in their expression. In addition, many of the PRL responsive genes identified contribute to inflammation, proliferation, and cell survival in inflamed joints [39-43]. Taken together, we have molecular evidence for autocrine PRL signaling in synoviocytes, and our results suggest that NR4A2 may contribute to synovial hyperplasia and inflammation by increasing PRL expression.

## Discussion

We previously reported that the orphan nuclear receptor NR4A2 induces cellular changes that contribute to synovial hyperplasia [10], and we therefore aimed to explore the underlying molecular mechanisms in the current study. We conducted a gene expression screen to identify novel transcriptional targets of NR4A2 in synoviocytes, and identified the immunomodulatory peptide hormone PRL as a target of NR4A2. PRL mRNA and protein are potently induced by ectopic NR4A2 (Figure 2) and endogenous NR4A receptors are required for PRL expression (Figure 3). We determined that NR4A2 targets a 238 bp region of the distal PRL promoter through a mechanism that requires a functional DNA binding domain (Figure 5). Furthermore, NR4A2-induced PRL may function in an autocrine manner to promote synovial hyperplasia and inflammation. In support of this, we determined that PRL regulates the expression of a subset of genes that contribute to inflammation, proliferation, and cell survival in inflamed joints (Figure 6). Our results provide the first evidence for NR4A2 regulation of PRL transcription in synoviocytes, and highlight a novel transcriptional pathway that may impact inflammatory joint diseases.

While PRL is primarily secreted from the anterior pituitary gland with critical functions in lactation and reproduction, this hormone is also expressed in a number of extrapituitary sites such as the brain, mammary gland, and immune system [19]. PRL has numerous immunomodulatory functions including stimulating cytokine expression, iNOS production, and T-cell proliferation [19]. In line with this, elevated PRL levels have been linked to autoimmune disorders such as systemic lupus erythematosus, systemic sclerosis, and RA [19]. In patients with RA, high levels of circulating PRL are correlated with disease activity and structural damage [20,21]. PRL is also present in synovial fluid, where levels are significantly higher in patients with RA versus OA [20]. Further supporting a role for PRL in arthritis pathogenesis, the dopamine agonist bromocriptine reduces systemic PRL production and has beneficial effects in patients with RA and in animal models of disease [22]. While clinical data suggest pathogenic roles for PRL in inflammatory arthritis, a mechanistic understanding of how PRL expression is regulated within inflamed joints is lacking.

To our knowledge, this study is the first to describe a transcriptional mechanism contributing to the expression of PRL in synoviocytes. However, several studies conducted in lymphocytes, endometrial stromal cells, and breast cancer cells provide an overview of PRL transcription in peripheral tissues [30,34,44-47]. Consistent with these studies, we confirmed that PRL transcription in synoviocytes is conferred by the activation of an alternative distal promoter located approximately 5 kb upstream from the start-site of transcription (Figure 4). Transcription factors such as CREB, CCAAT/enhancer-binding protein (C/EBP), Pit-1, FOXO1A, AP-1 and Ets interact with the distal PRL promoter and induce transcription in a cell-type specific manner [29,44-49]. Of particular interest to the current study, an NR4A2 homolog, NR4A1 (Nur77 / NGFI-B), has recently been described as an inducer of the distal PRL promoter in endometrial stromal cells [47].

Through deletional analysis, we mapped the NR4A2 responsive region of the distal PRL promoter to a 238 bp sequence spanning −270 to -32 bp (Figure 5). NR4A2 appears to interact with this promoter sequence, since a well-characterized point mutation in the DNA binding domain (C283G) abrogates transcription. NR4A target genes can be regulated by receptor binding to NBRE sequences in promoters [14], however the PRL promoter appears to lack this element. Likewise, MMP-13 and IL-8 are induced by NR4A2 in synoviocytes via NBRE-independent mechanisms [10,13]. While NR4A2 may target a novel *cis*-element, there is also evidence that this receptor works in concert with other transcription factors to regulate gene expression. For example, IL-8 is potently induced by NR4A2 through interactions with p65, and MMP-1 is repressed by NR4A2 via negative interactions with Ets transcription factors [9,13]. While these protein-protein mechanisms are likely promoter and cell-type specific, NR4A2 may employ a similar mechanism on the PRL promoter. The NR4A2-responsive region of the PRL promoter contains binding sites for Ets1, Hox, FOXO1A, C/EBPβ, and NFκB transcription factors [34,48], and NR4A2 may interact with one or more of these factors to transactivate the promoter. Alternatively, NR4A2 may act further upstream of the PRL promoter by inducing expression of one of these transcription factors that subsequently transactivates the PRL promoter.

Within inflamed joints, resident synoviocytes and chondrocytes, and infiltrating immune cells are equipped to respond to PRL via activation of cell-surface PRL-R and intracellular JAK/STAT signaling pathways [23,25,26]. PRL likely mediates its effects *in vivo* by triggering a combination of autocrine and paracrine responses. Locally produced PRL can induce synoviocyte proliferation and upregulate the expression of IL-6, IL-8, and MMP-3 [23]. However, in our experiments, we did not observe these effects on proliferation or gene expression. Since the primary synoviocytes used for these experiments were derived from patients with RA, the different responses noted could be due in part to patient heterogeneity. We did confirm PRL-R expression, suggesting that the basic mechanism to respond to PRL was intact in our cells (Figure 6A). Furthermore, we documented a subset of 11 genes that are regulated by rhPRL in synoviocytes (Figure 6B). Importantly, Akt1, IRF1, and SOCS-1 are known transcriptional targets of PRL [36-38], and we demonstrated a 2-fold increase in their expression. In mammary epithelial cells, PRL induces the phosphorylation of STAT5, which in turn regulates Akt1 transcription and promotes cell survival [36]. In synoviocytes from patients with RA, this signaling molecule also plays an important role in cell survival and proliferation [39,40]. Interferon regulatory factor-1 (IRF-1) is an immediate-early gene induced by PRL in T-cells, where it has a regulatory role in cell growth [37]. Interestingly, IRF-1 is highly expressed in RA synovial tissue, suggesting an important role for this factor in disease [42]. The cell cycle inhibitor CDKN1A (CIP1/p21) was potently suppressed by PRL (1394-fold), consistent with reduced expression in RA synovial tissue correlating with enhanced synoviocyte migration and invasion [41]. The most potently induced genes, interferon-induced protein IFI-15 K (ISG15), interleukin 4 receptor (IL4R), and erythropoietin receptor (EPOR) have not been described previously as PRL target genes, suggesting that PRL regulates a unique set of genes in synoviocytes. Taken together, the PRL responsive genes identified in this experiment contribute to inflammation, proliferation, and cell survival [36-43]. Based on these molecular findings, we hypothesize that NR4A2-induced PRL contributes to synovial hyperplasia and inflammatory processes via the regulation of downstream target genes.

While PRL appears to have autocrine effects on synoviocytes, it is also likely that PRL exerts paracrine effects on other cell-types present in joints. PRL inhibits the apoptosis of chondrocytes induced by inflammatory cytokines and serum starvation [25,27], suggesting that PRL may have chondroprotective effects. Furthermore, PRL induces the growth and chondrogenic differentiation of bone marrow derived mesenchymal stem cells, supporting a role for this signaling pathway in cartilage formation and repair [26]. PRL may also target endothelial cells and regulate angiogenesis [50,51], a process that is enhanced in chronically inflamed joints. However, PRL can be cleaved into an anti-angiogenic form by MMPs released from chondrocytes [24], suggesting that these effects on angiogenesis are tightly regulated. Taken together, NR4A2-induced PRL may exert multiple effects in inflamed joints and it will be critical to explore the *in vivo* impact of this novel transcriptional pathway in future studies.

## Conclusions

We have identified PRL as a transcriptional target of NR4A2 in synoviocytes, providing new insight into the regulation of this gene in extrapituitary sites. Further investigation of the NR4A2-PRL pathway may lead to new treatments for inflammatory arthritis. In addition, the ability to regulate PRL levels with selective NR4A2 antagonists may provide novel approaches to manage hyperprolactinemia associated with other autoimmune disorders and conditions.

## Additional files

Additional file 1: Table S1. Gene expression data from K4IM cells transduced with NR4A2. Additional file 2: Figure S1. Effects of rhPRL on synoviocyte proliferation. Additional file 3: Table S2. Gene expression data from K4IM cells treated with rhPRL.

## Abbreviations

AP-1: Activator protein 1
AKT1: Ak strain transforming 1
BCL2L1: BCL-2-like protein 1
CDKN1A: Cyclin dependent kinase inhibitor 1A
C/EBP: CCAAT/enhancer binding protein
C/EBPD: CCAAT/enhancer binding protein delta
CREB: cAMP response element binding protein
ETS: E26 transformation-specific
EPOR: Erythropoietin receptor
FOXO1A: Forkhead box protein O1A
HFLS: Human fibroblast-like synoviocytes
HOX: Homeobox
IL4R: Interleukin 4 receptor
IL-8: Interleukin 8
IRF1: Interferon regulatory factor 1
IRF9: Interferon regulatory factor 9
ISG15: Interferon-induced protein IFI-15 K/Ubiquitin-like modifier
JAK/STAT: Janus kinase/signal transducers and activators of transcription
MMP: Matrix metalloproteinase
NBRE: NR4A binding response element
NFκB: Nuclear factor kappa B
NGFIB: Nerve growth factor IB
NR4A2: Nuclear receptor 4A2
OA: Osteoarthritis
PGE_2_: Prostaglandin E2
Pit-1: Pituitary specific positive transcription factor 1
PRL: Prolactin
PRL-R: Prolactin receptor
PsA: Psoriatic arthritis
RA: Rheumatoid arthritis
shRNA: small hairpin RNA
SH2B1: SH2B adapter protein 1
SOCS1: Suppressor of cytokine signaling 1
TEK: Tyrosine kinase endothelial, TIE-2
TIMP: tissue inhibitor of matrix metalloproteinase
TNF-α: Tumor necrosis factor alpha
