# Supplementary material for: Orphan nuclear receptor NR4A2 induces transcription of the immunomodulatory peptide hormone prolactin
Source: J Inflamm (Lond). 2015 Feb 18;12:13. doi: 10.1186/s12950-015-0059-2 (PMC4339243; doi:10.1186/s12950-015-0059-2)
Supplement: Additional file 1: Table S1. — Gene expression data from K4IM cells transduced with NR4A2. [file 12950_2015_59_MOESM1_ESM.pdf]

Supplemental Table 1. Gene expression data from K4IM cells transduced with NR4A2

Array Position	Gene Name	Control, Ct	NR4A2, Ct	Fold change
A1	18S-Hs99999901_s1	20.99	20.98	reference
A2	GAPDH-Hs99999905_m1	19.97	18.97	reference
A3	HPRT1-Hs99999909_m1	24.96	23.94	reference
A4	GUSB-Hs99999908_m1	25.94	24.94	reference
A5	FGA-Hs00241027_m1	Undetermined	37.07	
A6	PLG-Hs00264877_m1	Undetermined	Undetermined	
A7	SERPINC1-Hs00166654_m1	35.97	35.95	0.507257
A8	PRL-Hs00168730_m1	37.02	29.94	68.123572
A9	MMP2-Hs00234422_m1	27.97	29.98	0.125495
A10	ANG;RNASE4-Hs02379000_s	33.95	32.95	1.003410
A11	ANGPT1-Hs00181613_m1	30.97	27.99	3.974345
A12	ANGPT2-Hs00169867_m1	37.09	Undetermined	
B1	CXCL12-Hs00171022_m1	37.05	37.03	0.508124
B2	EDIL3-Hs00174781_m1	29.97	28.96	1.006749
B3	EPHB2-Hs00362096_m1	30.97	32.97	0.125237
B4	FGF1-Hs00265254_m1	28.95	30.96	0.124342
B5	FGF2-Hs00266645_m1	26.97	26.97	0.502696
B6	FGF4-Hs00173564_m1	Undetermined	Undetermined	
B7	FST-Hs00246256_m1	30.97	26.98	8.012268
B8	HGF-Hs00300159_m1	37.08	36.96	0.543901
B9	IL8-Hs00174103_m1	34.94	31.95	4.001174
B10	LEP-Hs00174877_m1	Undetermined	Undetermined	
B11	MDK-Hs00171064_m1	25.97	25.98	0.497683
B12	TYMP-Hs00157317_m1	Undetermined	Undetermined	
C1	PDGFB-Hs00234042_m1	30.98	33.98	0.062839
C2	PTN-Hs00383235_m1	31.96	31.95	0.506322
C3	PROK1-Hs00260905_m1	37.05	37.04	0.505828
C4	TGFA-Hs00608187_m1	37.15	37.01	0.552665
C5	TGFB1-Hs99999918_m1	25.97	25.97	0.501807
C6	TNF-Hs00174128_m1	37.03	Undetermined	
C7	VEGFA-Hs00900054_m1	30.98	29.97	1.010933
C8	VEGFB-Hs00173634_m1	25.95	25.96	0.497744
C9	VEGFC-Hs00153458_m1	30.96	29.97	1.003016
C10	CTGF-Hs00170014_m1	24.94	24.93	0.507293
C11	FBLN5-Hs00197064_m1	34.99	28.98	32.273701
C12	THBS1-Hs00962914_m1	24.94	20.94	8.073195
D1	TNFSF15-Hs00270802_s1	36.97	37.01	0.487463
D2	ITGA4-Hs00168433_m1	30.96	29.95	1.017138
D3	IFNB1-Hs01077958_s1	36.97	36.98	0.497366
D4	IFNG-Hs00174143_m1	Undetermined	Undetermined	
D5	CXCL10-Hs00171042_m1	37.08	37.05	0.511025
D6	IL12A-Hs00168405_m1	33.96	34.97	0.250256
D7	SERPINF1-Hs00171467_m1	37.03	36.00	1.021844
D8	PF4-Hs00427220_g1	Undetermined	Undetermined	
D9	VASH1-Hs00208609_m1	33.98	32.98	1.001817
D10	ADAMTS1-Hs00199608_m1	28.97	29.96	0.254286
D11	ANGPTL1-Hs00559786_m1	36.98	37.14	0.449213
D12	AMOT-Hs00611096_m1	34.96	34.97	0.500919
E1	CD44-Hs00153304_m1	25.97	25.95	0.507280
E2	CDH5-Hs00174344_m1	35.97	32.97	4.020906
E3	CXCL2-Hs00601975_m1	32.97	32.97	0.504075
E4	SERPINB5-Hs00184728_m1	36.98	37.03	0.486185
E5	FLT1-Hs00176573_m1	Undetermined	37.06	
E6	SEMA3F-Hs00188273_m1	35.97	37.03	0.240404
E7	TEK-Hs00176096_m1	35.94	31.95	8.016538
E8	TIE1-Hs00178500_m1	Undetermined	Undetermined	
E9	TNMD-Hs00223332_m1	Undetermined	Undetermined	
E10	TIMP2-Hs00234278_m1	25.98	25.97	0.502656
E11	TIMP3-Hs00165949_m1	35.95	35.96	0.499131
E12	ANGPTL2-Hs00765775_m1	29.96	29.95	0.503725
F1	ANGPTL3-Hs00205581_m1	37.05	Undetermined	
F2	CEACAM1-Hs00236077_m1	36.96	37.09	0.458249
F3	HEY1-Hs00232618_m1	35.99	35.98	0.505067
F4	ITGAV-Hs00233808_m1	27.95	26.97	0.989899
F5	PECAM1-Hs00169777_m1	37.24	37.10	0.553242
F6	LYVE1-Hs00272659_m1	Undetermined	Undetermined	
F7	KIT-Hs00174029_m1	Undetermined	Undetermined	
F8	TNNI1-Hs00913333_m1	35.94	36.98	0.244309
F9	NRP2-Hs00187290_m1	28.95	27.95	1.010869
F10	KDR-Hs00176676_m1	Undetermined	Undetermined	
F11	ENPP2-Hs00196470_m1	30.97	29.96	1.009897
F12	FIGF-Hs00189521_m1	35.95	33.95	2.010070
G1	FOXC2-Hs00270951_s1	29.98	29.97	0.506095
G2	COL4A1-Hs00266237_m1	33.99	33.99	0.502362
G3	COL4A2-Hs01098873_m1	25.95	25.96	0.499355
G4	COL15A1-Hs00266332_m1	Undetermined	Undetermined	
G5	HSPG2-Hs00194179_m1	32.98	33.98	0.249770
G6	COL18A1-Hs00181017_m1	36.97	Undetermined	
G7	FN1-Hs01549940_m1	27.96	25.95	2.027555
G8	COL4A3-Hs01022527_m1	Undetermined	Undetermined	
G9	F2-Hs01011995_g1	34.97	34.95	0.507449
G10	BAI1-Hs01105174_m1	Undetermined	Undetermined	
G11	CHGA-Hs00900373_m1	37.02	Undetermined	
G12	ANGPT4-Hs00211115_m1	Undetermined	Undetermined	
H1	CSF3-Hs99999083_m1	Undetermined	Undetermined	
H2	GRN-Hs00963711_g1	23.97	23.98	0.499637
H3	THBS2-Hs01568063_m1	28.99	28.96	0.512394
H4	LECT1-Hs00993254_m1	Undetermined	Undetermined	
H5	ANGPTL4-Hs01101127_m1	33.97	33.97	0.504320
H6	ITGB3-Hs01001469_m1	34.96	31.98	3.978366
H7	PDGFRA-Hs00998026_m1	35.96	35.96	0.502685
H8	PDGFRB-Hs00387364_m1	30.96	33.96	0.062885
H9	FLT4-Hs01047677_m1	34.98	33.98	1.009024
H10	NRP1-Hs00826128_m1	27.96	26.95	1.005750
H11	S1PR1-Hs01922614_s1	27.94	26.94	1.004412
H12	PROX1-Hs00896294_m1	37.02	35.96	1.049121
