# Supplementary material for: Orphan nuclear receptor NR4A2 induces transcription of the immunomodulatory peptide hormone prolactin
Source: J Inflamm (Lond). 2015 Feb 18;12:13. doi: 10.1186/s12950-015-0059-2 (PMC4339243; doi:10.1186/s12950-015-0059-2)
Supplement: Additional file 2: Figure S1. — Effects of rhPRL on synoviocyte proliferation. [file 12950_2015_59_MOESM2_ESM.pdf]

Supplemental Figure 1. Effects of rhPRL on synoviocyte proliferation

A

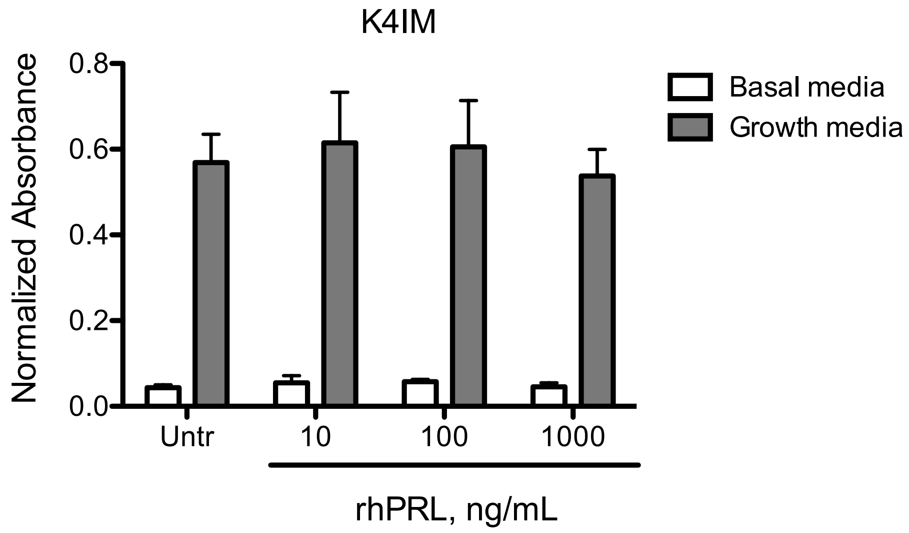

B

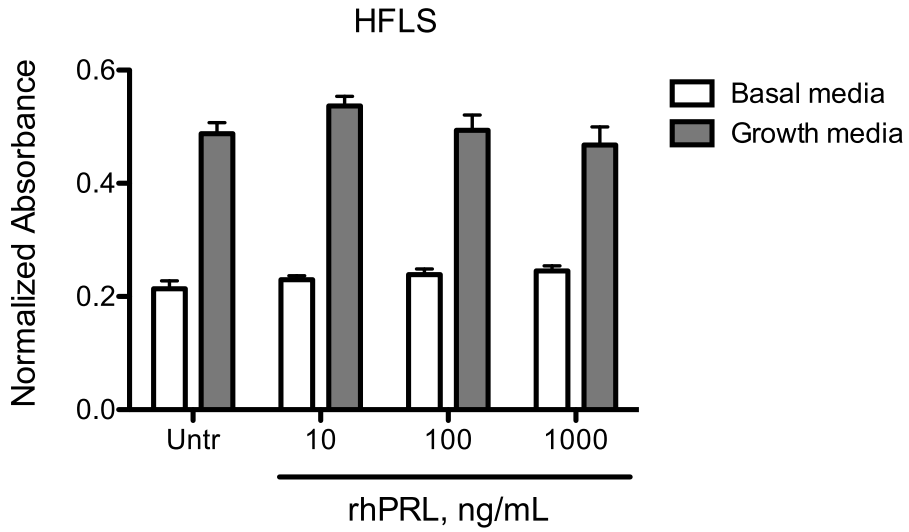

K4IM synoviocytes (A) and RA-HFLS (B) were cultured in serum-free basal media or growth media containing 10% FBS alone or with rhPRL (10-1000ng/mL) for 96 hours. Cell growth and viability was measured using the Promega CellTiter 96 cell proliferation assay and absorbance at 570nm was recorded.
