# Supplementary material for: Orphan nuclear receptor NR4A2 induces transcription of the immunomodulatory peptide hormone prolactin
Source: J Inflamm (Lond). 2015 Feb 18;12:13. doi: 10.1186/s12950-015-0059-2 (PMC4339243; doi:10.1186/s12950-015-0059-2)
Supplement: Additional file 3: Table S2. — Gene expression data from K4IM cells treated with rhPRL. [file 12950_2015_59_MOESM3_ESM.pdf]

Supplemental Figure 2. Gene expression data from K4IM cells treated with rhPRL

Position	Symbol	Untreated, Ct	rhPRL, Ct	Fold Regulation
A01	A2M	35	35	-1.1456
A02	AKT1	26.04	24.77	2.094
A03	BCL2L1	27.25	26.04	2.0145
A04	CCND1	26.56	25.76	1.5247
A05	CDKN1A	24.75	35	-1394.7208
A06	CEBPB	30.57	29.73	1.5587
A07	CEBPD	30.19	28.99	2.0131
A08	CRK	27.53	26.94	1.3104
A09	CRP	35	35	-1.1456
A10	CSF1R	35	35	-1.1456
A11	CXCL9	35	35	-1.1456
A12	EGFR	26.43	25.53	1.6247
B01	EPOR	35	30.5	19.7081
B02	F2	35	34.19	1.5334
B03	F2R	33.97	33.26	1.4295
B04	FAS	27.98	27.68	1.0749
B05	FCER2	35	35	-1.1456
B06	FCGR1A	35	35	-1.1456
B07	GATA3	32.26	31.78	1.2151
B08	GHR	35	35	-1.1456
B09	GRB2	28.11	27.15	1.7008
B10	IFNAR1	26.6	25.6	1.7408
B11	IFNG	35	35	-1.1456
B12	IFNGR1	28.75	28.49	1.0417
C01	IL10RA	35	35	-1.1456
C02	IL20	35	35	-1.1456
C03	IL2RA	35	35	-1.1456
C04	IL2RG	35	35	-1.1456
C05	IL4	35	35	-1.1456
C06	IL4R	35	29.33	44.5096
C07	IL6ST	25.85	24.74	1.877
C08	INSR	31.57	30.37	1.9965
C09	IRF1	28.38	26.99	2.2855
C10	IRF9	27.4	26.16	2.0661
C11	ISG15	35	26.6	295.1941
C12	JAK1	25.84	25.29	1.2767
D01	JAK2	30	29.57	1.1788
D02	JAK3	35	35	-1.1456
D03	JUN	28.46	27.87	1.3081
D04	JUNB	31.16	30.33	1.5535
D05	LRG1	35	35	-1.1456
D06	MCL1	23.91	23.44	1.2043
D07	MPL	35	35	-1.1456
D08	MYC	26.71	26.08	1.3453
D09	NFKB1	29.08	28.2	1.6107
D10	NOS2	35	35	-1.1456
D11	NR3C1	26.68	25.73	1.6856
D12	OAS1	30.72	29.55	1.9608
E01	OSM	35	35	-1.1456
E02	PDGFRA	35	35	-1.1456
E03	PIAS1	26.85	26.15	1.4238
E04	PIAS2	27.92	27.35	1.2986
E05	PIAS3	26.11	26.19	-1.2127
E06	PRL	35	35	-1.1456
E07	PRLR	34.11	34.11	-1.1382
E08	PTPN1	28.39	28.15	1.0342
E09	PTPN11	27.24	26.52	1.4376
E10	PTPRC	35	35	-1.1456
E11	SH2B1	28.37	26.99	2.27
E12	SMAD1	29.23	28.68	1.2802
F01	SMAD2	26.56	25.8	1.4699
F02	SMAD3	32.12	31.63	1.2206
F03	SMAD4	27.41	27	1.1539
F04	SMAD5	27.44	27	1.1819
F05	SOCS1	33.15	31.47	2.7988
F06	SOCS2	31.91	31.06	1.5698
F07	SOCS3	31.37	31.04	1.0928
F08	SOCS4	27.61	27.22	1.1407
F09	SOCS5	27.25	27.01	1.0306
F10	SP1	26.89	26.19	1.4154
F11	SPI1	35	35	-1.1456
F12	SRC	29.34	28.5	1.5625
G01	STAM	27.37	27.34	-1.1186
G02	STAT1	25.61	25.04	1.295
G03	STAT2	27.29	26.16	1.9096
G04	STAT3	26.03	25.24	1.5111
G05	STAT4	32.42	31.77	1.3662
G06	STAT5A	35	34.03	1.707
G07	STAT5B	27.8	27.55	1.0347
G08	STAT6	27.13	26.81	1.0846
G09	STUB1	26.25	26.3	-1.1883
G10	TYK2	28.84	28.36	1.2137
G11	USF1	29.11	28.28	1.5516
G12	YY1	26.08	25.48	1.3254
H01	ACTB	20.13	19.23	1.6223
H02	B2M	21.74	21.51	reference
H03	GAPDH	20.52	20.22	reference
H04	HPRT1	26.11	25.22	1.6206
H05	RPLP0	20.29	20.22	reference
H06	HGDC	35	35	-1.1456
H07	RTC	25.3	26.05	-1.9306
H08	RTC	25.21	25.96	-1.9254
H09	RTC	25.11	26	-2.1104
H10	PPC	21.63	21.23	1.1551
H11	PPC	21.59	35	-12451.8481
H12	PPC	22.03	35	-9191.2064
